# Quantum Thermalization and the Expansion of Atomic Clouds

**DOI:** 10.1038/s41598-017-06193-0

**Published:** 2017-07-21

**Authors:** Louk Rademaker, Jan Zaanen

**Affiliations:** 1 Kavli Institute for Theoretical Physics, University of California, Santa Barbara, CA 93106 USA; 2 Institute-Lorentz for Theoretical Physics, Leiden University, P.O. Box 9506, Leiden, The Netherlands

## Abstract

The ultimate consequence of quantum many-body physics is that even the air we breathe is governed by strictly unitary time evolution. The reason that we perceive it nonetheless as a completely classical high temperature gas is due to the incapacity of our measurement machines to keep track of the dense many-body entanglement of the gas molecules. The question thus arises whether there are *instances where the quantum time evolution of a macroscopic system is qualitatively different from the equivalent classical system*? Here we study this question through the expansion of noninteracting atomic clouds. While in many cases the full quantum dynamics is indeed indistinguishable from classical ballistic motion, we do find a notable exception. The subtle quantum correlations in a Bose gas approaching the condensation temperature appear to affect the expansion of the cloud, as if the system has turned into a diffusive collision-full classical system.

## Introduction

The laws describing classical gases, most notably the Second Law of Thermodynamics, seem at odds with the principle of unitary time evolution in quantum physics^1^. However, high energy states are densely many-body entangled and consequently the Eigenstate thermalization hypothesis (ETH)^2–4^ claims that the outcomes of local measurements will be at long times indistinguishable from the outcome of the measurement in a thermal mixed state, at a temperature consistent with the energy of that state^5, 6^.

Is this also true for a cloud of non-interacting quantum particles confined in a potential, which is suddenly released and allowed to expand in an infinite bath? This is actually similar to the key ‘time-of-flight measurement’ in many cold atom experiments^7, 8^. After suddenly releasing the confining potential the atomic clouds expand, and by assuming that this is governed by ballistic, collision-less atomic motion the initial velocity distributions can be deduced from the expansion of the cloud. Invariably, it has been assumed that this expansion is governed by a purely classical Newtonian or wave kinematics, and at first sight this seems the correct procedure to follow.

**Figure 1:**
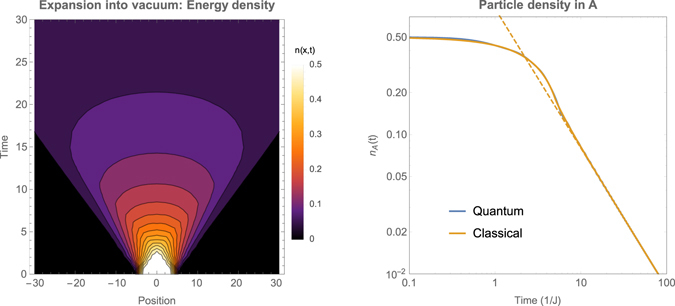
Expansion of an atomic cloud into the vacuum. Initially we prepare an one-dimensional atomic cloud in region *A* (|*x*| < 5), at inverse temperature *β* = 0.01, with particle density *n* = 0.5. On the left we show the energy density as a function of position *x* and time *t*, following exact quantum evolution. On the right we show the density of particles *n*_*A*_(*t*) in region *A*. The full quantum evolution is correctly represented by a classical distribution of particle positions and velocities *n*(*x*, *v*, *t*) whose time evolution is given by ballistic motion, *n*(*x*, *v*, *t*) = *n*(*x* − *vt*, *v*, 0). At late times, the particle density in region *A* decays as *n*_*A*_ ~ 1/*t*, in the right graph shown as a dashed line.

However, it is not at all obvious why this works. After all, before releasing the trapping potential, one may be in a quantum regime with Bose condensation or Fermi-degeneracy. How can these atoms suddenly behave like classical canon balls? In the next section, we will present a method to compute local observables exactly in the full quantum evolution by evaluating the logarithm of the density matrix. Our first result, of a particle cloud released into the vacuum, is shown in Fig. 1: under the conditions of the cold atom experiments the full quantum dynamics is indeed *indistinguishable* from classical ballistic expansion.

**Figure 2:**
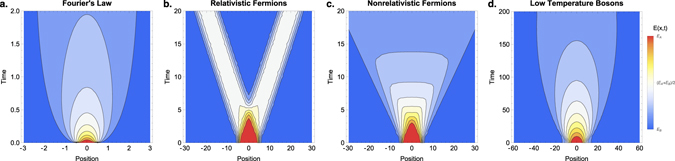
The energy spread of an initial subsystem *A* at a hot temperature *T*_*A*_ (in red) immersed in a cold bath at *T*_*B*_ (in blue), for four different theories, computed in *d* = 1. For the right three panels we performed a lattice computation with *L* = 200 and *L*_*A*_ = 10, so that region *A* is defined as 5 < *x* ≤ 5. The colors represent local energy density, and interpolates linearly between *E*_*A*_ and *E*_*B*_. (**a**) The classical Fourier’s law predicts a diffusive spread of the heat, here computed using the heat kernel with region *A* defined as |*x*| < 0.5. The temperature difference in subsystem *A* vanishes diffusively according to Δ*T*(*t*) ~ *t* ^−1/2^. (**b**) For relativistic fermion systems (*T*_*A*_ = 2, *T*_*B*_ = 1, *n* = 0.5) there is instantaneous thermalization once *A* is in complete causal contact with the bath. (**c**) In non-relativistic fermion systems (*T*_*A*_ = 2, *T*_*B*_ = 1, *n* = 0.5) there is ballistic transport of particles, however, since not all particles have the same speed there is a power-law decay of the initial temperature difference, following Δ*T*(*t*) ~ *t* ^−1^. (**d**) Non-relativistic boson systems display a crossover from ballistic to diffusive thermalization. Here we show the energy profile in the low temperature regime where diffusive behavior is visible, *T*_*A*_ = 100, *T*_*B*_ = 0.2 and *n* = 0.5.

**Figure 3:**
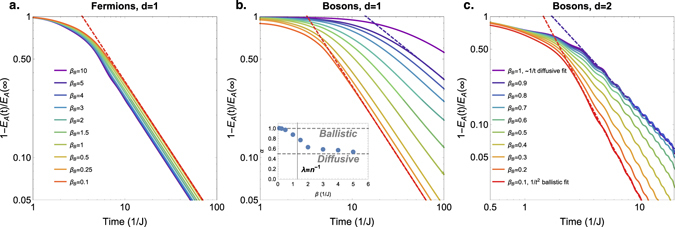
Decay of the energy difference between the system and the bath in non-relativistic fermionic or bosonic systems. In *d* = 1 (left two pictures) we have immersed a subsystem *A* at almost infinite temperature *β*_*A*_ = 0.01 in a bath with varying temperatures *β*_*B*_. The chemical potential is tuned such that the particle density is *n* = 1/2, and the total system size is *L* = 200, and *L*_*A*_ = 10. On the vertical axis we plot the energy density in subsystem *A*, *E*_*A*_(*t*), normalized by the energy density at infinite time *E*_*A*_(*t* = ∞). In *d* = 2 (right picture) the subsystem *A* has size *N*_*A*_ = 6 × 6 in a total system size of *N* = 48 × 48. (**a**) In fermionic systems, the decay is always of a ballistic nature, Δ*E* ~ *t* ^−*d*^. (**b**) In bosonic systems, there is a crossover from ballistic *t* ^−*d*^ to diffusive *t* ^−*d*/2^ decay. We fit the long-time behavior with the power-law form *t* ^−*α*^. The inset shows the power *α* as a function of inverse bath temperature *β*_*B*_. The crossover from ballistic to diffusive occurs around the point *β*_*c*_ ~ 1.3 where the thermal de Broglie wavelength *λ* is comparable to the interparticle spacing *n* ^−1^, suggesting the wave-like nature of the bosons is responsible for the diffusive behavior. (**c)** The crossover can also be observed in *d* = 2 dimensions. The crossover occurs at higher temperatures, since the value where *λ* ~ *n* ^−1/2^ has shifted to higher temperature, *β*_*c*_ ~ 0.8. Small oscillations with period 1/4 *J* can be observed due to the specific choice of lattice dispersion.

We then address cooling, where the atoms are released in a particle bath which is at a lower temperature than the trapped particles. When the temperature of the bath is high enough we find an expansion consistent with the classical expectation: since the particles do not collide, the hot cloud cools ballistically. Similarly, when the cloud and the bath are both formed from fermions the system behaves classical at all temperatures. However, for a cloud of bosons cooling into a bosonic bath at a temperature approaching the condensation temperature, the cooling is governed by *diffusion*! In Fig. 2 we show how the energy density of a ‘hot’ cloud in a cold bath spreads out in time, marking a clear difference between classical diffusion, ballistic fermionic behavior and again diffusion for a low temperature bosonic bath. Quantitatively, the difference between ballistic and diffusive behavior can be shown by measuring the total energy density Δ*E* in the region of the original cloud relative to the bath energy density, as shown in Fig. 3: ballistic decay is characterized by Δ*E* ~ *t* ^−*d*^ whereas diffusion satisfies Δ*E* ~ *t* ^−*d*/2^.

This is our main result. We have identified a circumstance where the quantum evolution becomes sharply distinguishable from the analogous classical evolution. In the classical system diffusional expansion requires collisions, but these are collision-less quantum particles. We will explain how to test this prediction in cold atom experiments, but first we elucidate how these matters are computed.

## Method

The traditional approach to evaluate quantum time evolution is by repeated application of the time evolution operator ${e}^{-i {\mathcal H} dt}$ with small temporal steps *dt*. However, with this procedure it is impossible to reach times later than *t* ~ 1/*E*, where *E* is a typical energy scale of the system. The hypothesis of thermalization, that is *ρ* → *e* ^−*βH*^ locally at late times, provides us now with a simpler way to compute time evolution through the *modular Hamiltonian* ${\mathcal M}$, which is the logarithm of the density matrix,

1 $$ {\mathcal M} =-\mathrm{log}\,\rho .$$

As we will see, at late times ${\mathcal M}$ will simplify dramatically. Since we are interested in a hot cloud in a cold bath, our initial density matrix will have the form

2 $${\rho }_{0}=\frac{1}{{Z}_{A}{Z}_{B}}{e}^{-{\beta }_{A}{ {\mathcal H} }_{A}}\otimes {e}^{-{\beta }_{B}{ {\mathcal H} }_{B}}$$

where ${ {\mathcal H} }_{X}$ and *β*_*X*_ are the total Hamiltonian and inverse temperatures respectively, restricted to the subsystems *X* = *A*, *B*. Note that this is equivalent, up to boundary terms, to ${\rho }_{0}\sim {{\rm{Tr}}}_{B}{e}^{-{\beta }_{A} {\mathcal H} }\otimes {{\rm{Tr}}}_{A}{e}^{-{\beta }_{B} {\mathcal H} }$. The time evolution of the modular Hamiltonian follows directly from the von Neumann equation for the time evolution of the density matrix,

3 $$ {\mathcal M} (t)={e}^{-i {\mathcal H} t}{ {\mathcal M} }_{0}{e}^{i {\mathcal H} t}.$$

For noninteracting systems ${\mathcal H} ={\sum }_{k}{\xi }_{k}{n}_{k}$, the initial modular Hamiltonian following from Eqn. (2) can be written as,

4 $${ {\mathcal M} }_{0}=\sum _{kk^{\prime} }{m}_{kk^{\prime} }{\hat{c}}_{k}^{\dagger }{\hat{c}}_{k^{\prime} }+\,\mathrm{log}\,Z.$$

The modular matrix $\hat{m}={m}_{kk^{\prime} }$ is Hermitian; the sum runs over the momenta *k* of the particles, while the constant $\mathrm{log}\,Z=-\eta {\rm{Tr}}\,\mathrm{log}(1-\eta {e}^{-\hat{m}})$, with *η* = −1 for fermions and *η* = +1 for bosons. The time evolution of both fermion and boson field operators appearing in the modular Hamiltonian is for the free system simply given by,

5 $${\hat{c}}_{k}^{\dagger }(t)={e}^{-i {\mathcal H} t}{\hat{c}}_{k}^{\dagger }{e}^{i {\mathcal H} t}={e}^{-i{\xi }_{k}t}{\hat{c}}_{k}^{\dagger },$$

This implies for the time dependence of the modular Hamiltonian,

6 $$ {\mathcal M} (t)=\sum _{kk^{\prime} }{m}_{kk^{\prime} }{e}^{-i({\xi }_{k}-{\xi }_{k^{\prime} })t}\,{\hat{c}}_{k}^{\dagger }{\hat{c}}_{k^{\prime} }+\,\mathrm{log}\,Z$$

7 $$\equiv \sum _{kk^{\prime} }{m}_{kk^{\prime} }(t)\,{\hat{c}}_{k}^{\dagger }{\hat{c}}_{k^{\prime} }+\,\mathrm{log}\,Z$$

It follows that time evolution corresponds with a unitary transformation on the modular matrix. Local observables as the occupation numbers and the energy are in turn functions of the equal-time Greens function at time *t*, ${G}_{ij}(t)={\rm{Tr}}\,{\hat{c}}_{i}^{\dagger }{\hat{c}}_{j}\rho (t)$, in terms of the modular matrix

8 $$\hat{G}(t)={[{e}^{\hat{m}(t)}-\eta ]}^{-1}.$$

The advantage of this formulation starts to shimmer through. The intricacies of the full quantum evolution are absorbed in the strongly oscillating factors occurring in Eq. (7). These will rapidly average away such that in the limit *t* → ∞, the modular Hamiltonian approaches the actual Hamiltonian, ${\mathcal M} (t)\to \beta  {\mathcal H}$ when expressed in a *local basis*.

### Expansion of a noninteracting hot gas in a cold bath

To see how this works let us consider some examples. Relativistic systems are discussed in the supplementary material, reproducing the wisdom that these thermalize instantaneously once full causal contact is established^9–12^. To model non-relativistic atoms we resort to a lattice regularization in the form of a hypercubic lattice in *d* dimensions with nearest neighbor hopping and periodic boundary conditions,

9 $$ {\mathcal H} =-J\sum _{{\bf{r}},\delta }({\hat{c}}_{{\bf{r}}+\delta }^{\dagger }{\hat{c}}_{{\bf{r}}}+{\hat{c}}_{{\bf{r}}}^{\dagger }{\hat{c}}_{{\bf{r}}+\delta })=\sum _{{\bf{k}}}{\varepsilon }_{k}{\hat{n}}_{k}$$

with ${\varepsilon }_{{\bf{k}}}=-2J{\sum }_{i=1}^{d}\,\cos \,{k}_{i}$. Given our initial hot cloud state the modular Hamiltonian equals ${\mathcal M} (t)={\beta }_{B} {\mathcal H} +({\beta }_{A}-{\beta }_{B}){ {\mathcal H} }_{A}(t)$ where the Hamiltonian of the subsystem *A* is at *t* = 0 equal to

10 $${ {\mathcal H} }_{A}=-J\sum _{{r}_{x},{r}_{y},\ldots =1}^{{L}_{A}-1}\sum _{\delta }({\hat{c}}_{{\bf{r}}+\delta }^{\dagger }{\hat{c}}_{{\bf{r}}}+{\hat{c}}_{{\bf{r}}}^{\dagger }{\hat{c}}_{{\bf{r}}+\delta }).$$

Under time evolution this hot cloud spreads out and at *t* > 0 we express ${\mathcal M} (t)=-J{\sum }_{j\ell }{m}_{j\ell }(t){\hat{c}}_{j}^{\dagger }{\hat{c}}_{\ell }$ in terms of the elements of the modular matrix ${m}_{j\ell }(t)$ in the real space basis,

11 $$\begin{array}{rcl}{m}_{j\ell }(t) & = & {\beta }_{B}{\delta }_{|j-\ell |=1}+({\beta }_{A}-{\beta }_{B})\int \frac{{d}^{d}{\bf{k}}{d}^{d}{\bf{k}}^{\prime} }{{\mathrm{(2}\pi )}^{2d}}{e}^{-i{\rm{\kappa }}{{\bf{r}}}_{j}+i{\rm{\kappa }}{{\bf{r}}}_{\ell }}(\sum _{i=1}^{d}{e}^{i{{\bf{k}}}_{i}}+{e}^{-i{{\bf{k}}}_{i}^{^{\prime} }})\\  &  & (\prod _{i=1}^{d}\frac{{e}^{i({k}_{i}-{k}_{i}^{^{\prime} })({L}_{A}-1)}-1}{{e}^{i({k}_{i}-{k}_{i}^{^{\prime} })}-1}){e}^{i({\varepsilon }_{{\rm{\kappa }}}-{\varepsilon }_{{\rm{\kappa }}^{\prime} })t}.\end{array}$$

Recall that thermalization in the ETH sense implies that the second term should vanish at late times. Indeed, using the continuum approximation *ε*_**k**_ ≈ *Jk* ^2^ − *μ* for *t* ≫ 1/*ε*_**k**_, and thus (**ε**_k_ − **ε**_k′_)*t* ≈ (k + k′) (k − k′)*Jt*, we find for a site *j* ∈ *A*,

12 $${\rm{\Delta }}{m}_{j,j+1}(t\gg 1)=2{\rm{\Delta }}\beta (0){(\frac{{L}_{A}-1}{2\pi Jt})}^{d}\sim \frac{{V}_{A}}{{t}^{d}}.$$

Regardless of the statistics of the particles, the modular Hamiltonian approaches the final thermal state with a ballistic powerlaw decay.

However, the experimentally relevant *local energy density* in subsystem *A* can approach the bath value in different manners, pending the quantum statistics of the particles as illustrated in Fig. 3. Fermions are consistently subjected to a ballistic decay of the energy difference between the bath and the subsystem *A* (Fig. 3a) and the resulting energy flow profile (Fig. 2c) displays a smoothened light-cone following the Lieb-Robinson bound with *v*_*LR*_ = 2 *J* ^13^. Turning to bosons, the surprise we announced becomes manifest: we find a crossover from ballistic behavior at high bath temperatures to diffusive Δ*E* ~ *t* ^−*d*/2^ at low bath temperatures. For both *d* = 1 and *d* = 2 dimensions (Fig. 3b,c), the crossover occurs around the point where the lattice thermal de Broglie wavelength corresponds to the interparticle spacing. This suggests that diffusive behavior is a consequence of the wave-like nature of the bosons, where ${\partial }_{t}\psi \sim {\partial }_{x}^{2}\psi$.

The energy profile of the diffusive case (Fig. 2d) is surprisingly reminiscent of the classical Fourier’s law of heat diffusion (Fig. 2a). However, one should not be fooled by this apparent relation to classical diffusion. After all, we are considering noninteracting particles and the equivalent classical description of our set-up is through a distribution of particles and velocities *n*(*x*, *v*, *t*) that evolves ballistically *n*(*x*, *v*, *t*) = *n*(*x* − *vt*, *v*, 0). For the expansion into a cold bosonic bath the classical picture still yields a ballistic spread, while the exact quantum evolution displays diffusive behavior. The diffusive behavior for cold bosonic baths is therefore a genuine quantum effect.

## Conclusion and Outlook

**Figure 4:**
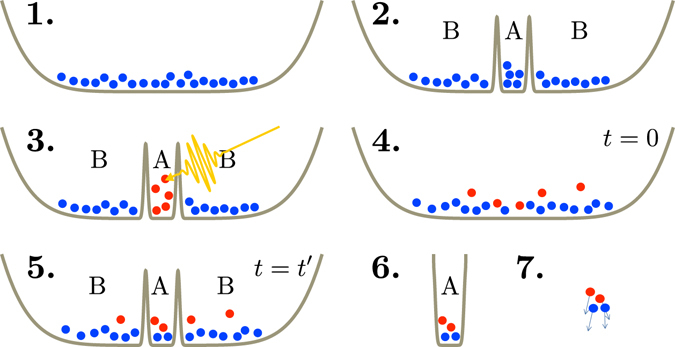
Cartoon of the suggested cold-atom experiment. 1. Prepare a trapped cloud of noninteracting atoms at a temperature *T*_*B*_. 2. Introduce a barrier that separates the system into *A* and a bath *B*. 3. Using a laser, heat up the atoms in region *A* to temperature *T*_*A*_. 4. At time *t* = 0 remove the barrier between *A* and *B*, letting the two subsystems thermalize. 5. After a time *t* = *t*′ reintroduce the barrier between *A* and *B*. 6. Remove the trap around region *B*. 7. Remove the trap around *A* and perform a time-of-flight measurement of the kinetic energy of the atoms in *A*. The steps 1–7 should be repeated for different times *t*′ to obtain the energy in *A* as a function of time after the quench.

The ballistic-to-diffusive crossover for low-temperature bosons as shown in Fig. 3b can be probed directly in experiments using cold atoms, following the protocol illustrated in Fig. 4 ^14–16^. Initially, one prepares a cloud of atoms tuned to be noninteracting using the Feshbach resonance. Using optical lattice techniques a barrier is created in between *A* and *B*, and a separate laser excites *A* to be at a different temperature than *B*. At time *t* = 0 the barrier is removed and the system will evolve as described. To measure the energy density in subsystem *A* after a time *t*, one reintroduces the barrier, let the atoms in the bath *B* escape, followed by time-of-flight measurements of the distribution of momenta of the atoms in *A*. From the distribution of these momenta the total kinetic energy can be reconstructed. The experiment is then repeated to obtain the energy density in *A* at every time instance. In this way the curves of Fig. 3, for either ballistic or diffusive behavior, can be experimentally measured.

It might be a surprise to observe thermalization in integrable non-interacting systems, but it is quite straightforward that this happens for local quenches such as the one studied here^17, 18^. Even though there are many integrals of motion, there is no conservation law that restricts certain degrees of freedom to remain within *A*, though now thermalization implies an approach to the Generalized Gibbs Ensemble rather than the standard Gibbs ensemble^4, 19–21^. Note, however, that systems where the integrals of motion are truly local, as is the case for Anderson insulators^22^ or the many-body localized phase^23, 24^, information remains within *A* and no thermalization will occur.

A critical reader might object that the system we study actually displays an entropy decrease. However, much like refrigerators, we reduce the entropy of subsystem *A* by increasing the bath entropy by at least the same amount. In fact, while the total entropy remains constant in any quantum system, the mutual information ${ {\mathcal I} }_{AB}(t)={S}_{A}(t)+{S}_{B}(t)-{S}_{A\cup B}$ increases upon thermalization since the subsystem *A* and the bath *B* become entangled. This increase in mutual information should be considered the quantum version of the Second Law^1^. However, it remains an open question to prove this increase for thermodynamically large systems as the Second Law requires.

## Electronic supplementary material

Supplementary information
