# Supplementary material for: Quantum Thermalization and the Expansion of Atomic Clouds
Source: Sci Rep. 2017 Jul 21;7:6118. doi: 10.1038/s41598-017-06193-0 (PMC5522402; doi:10.1038/s41598-017-06193-0)
Supplement: Supplementary file 1 — Supplementary information [file 41598_2017_6193_MOESM1_ESM.pdf]

Supplementary Information for 'Quantum Thermalization and the Expansion of Atomic Clouds'

Louk Rademaker[1,*] and Jan Zaanen[2]

[1]Kavli Institute for Theoretical Physics, University of California Santa Barbara, CA 93106, USA
[2]Institute-Lorentz for Theoretical Physics, Leiden University, P.O. Box 9506, Leiden, The Netherlands
[*]louk.rademaker@gmail.com

ABSTRACT

This supplementary information contains background, methods and a few additional results pertaining to the main manuscript '*Eigenstate thermalization and the expansion of atomic clouds*'.

Thermalization of classical systems - Fourier's Law

In the main manuscript we consider a hot system A at temperature T_A immersed in a cold bath at temperature T_B. How will thermal equilibrium be reached according to the classical theory of thermal diffusion? There it is assumed that a system is locally in thermal equilibrium, such that one can define a temperature $T(x)$ at each point in space. If there exists a temperature gradient, energy will flow from hot to cold according to Fourier's law,

$$j_E(x) = -\kappa(T(x))\,\nabla T(x) \tag{A.1}$$

where κ is the thermal conductivity of the material and $j_E(x)$ is the energy current. If we are in a regime where both the specific heat c_V and the thermal conductivity κ are approximately independent of temperature, Fourier's Law becomes a diffusion equation

$$\partial_t T = \mathscr{D}\nabla^2 T. \tag{A.2}$$

where the diffusion constant is $\mathscr{D} = \frac{\kappa}{c_V}$. This equation can be solved using the heat kernel.

Let us look explicitly at an initial state with a hot cloud at temperature T_A for $|x| < a/2$, and and a bath at T_B for $|x| > a/2$. The resulting solution of the heat diffusion equation yields

$$\Delta T(x,t) = \frac{1}{2}(T_A - T_B)\left(\mathrm{Erf}\left[\frac{a-2x}{4\sqrt{\mathscr{D}t}}\right] + \mathrm{Erf}\left[\frac{a+2x}{4\sqrt{\mathscr{D}t}}\right]\right). \tag{A.3}$$

In the left panel of Fig. 1 in the main manuscript, we show how the heat of our cloud spreads according to the above formula.

Note that the temperature difference at long times falls of in a power law fashion, $\Delta T(x=0, t \gg 1) \sim \frac{a(T_A - T_B)}{2\sqrt{\pi\mathscr{D}t}}$. In higher dimensions d, the above equations straightforwardly generalize to

$$\Delta T(x=0, t \gg 1) \sim (T_A - T_B)\left(\frac{a}{2\sqrt{\pi\mathscr{D}t}}\right)^d \sim \frac{V_A}{t^{d/2}}. \tag{A.4}$$

Therefore, if the energy or temperature of a system decays as a powerlaw $t^{-d/2}$, we call this diffusion.

Comparison between classical and quantum description

Now consider another classical system: a gas of collision-less non-interacting particles. At time $t = 0$, we can characterize this gas as having a distribution of particles in position and velocity, $n(x, v, 0)$. The particle density as a function of position is $n(x) = \int dv\, n(x, v, 0)$, and with an energy per particle that depends only on velocity, $\varepsilon(v)$, the energy density is given by $E(x) = \int dv\, \varepsilon(v) n(x, v, 0)$.

Because the particles are collision-less and have no further interactions, the velocity is conserved. This means that the full distribution at a later time t can be expressed in terms of the initial distribution as

$$n(x, v, t) = n(x - vt, v, 0). \tag{A.5}$$

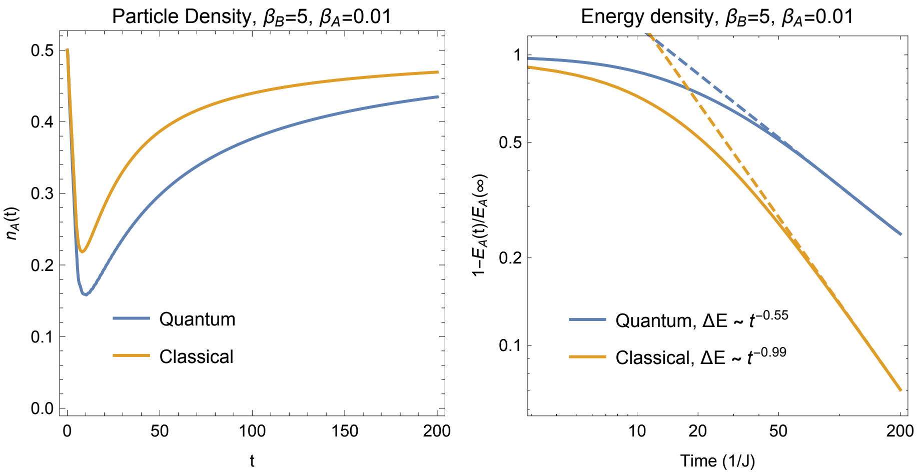

Figure A.1. Comparison between quantum and classical description of the expansion of a hot $\beta_A = 0.01$ boson system into a cold bath $\beta_B = 5$, with $L_A = 10$ and $n = 0.5$. The quantum description is slower and tends to diffusive behavior $\Delta E \sim t^{-1/2}$, whereas the classical description incorrectly predicts ballistic behavior.

To classically model a generic system of bosons, we can start with an initial distribution

$$n(x,v,0) = \frac{1}{2\pi}\frac{dk}{dv}\frac{1}{e^{\beta(x)(\varepsilon(v)-\mu(x))}-1} \tag{A.6}$$

with $\varepsilon_{\mathbf{k}}$ the boson dispersion and $v_{\mathbf{k}} = \frac{d\varepsilon_{\mathbf{k}}}{d\mathbf{k}}$. Note that the energy per particle is $\varepsilon_{\mathbf{k}} - \mu$. The initial temperature imbalance is characterized by a spatially varying inverse temperature $\beta(x)$ and chemical potential $\mu(x)$.

In the main manuscript we first considered a gas expanding into the vacuum. We model this in $d = 1$ by taking $n(x,v,0) = \frac{m}{2\pi}\frac{1}{e^{\beta(mv^2/2-\mu)}-1}$ when $x \in A$, and zero outside A. For bosons $\mu < 0$, so let's define $\alpha = e^{-\beta\mu} > 1$. The particle density at $x = 0$ at late time $t \gg 1$ then equals

$$\begin{aligned} n(x=0,t) &= 2\int_0^{L_A/2t} dv \frac{m}{2\pi}\frac{1}{e^{\beta(mv^2/2-\mu)}-1} && \text{(A.7)}\\ &\approx \frac{m}{\pi}\int_0^{L_A/2t} dv \frac{1}{(\alpha-1)+\beta mv^2/2} && \text{(A.8)}\\ &\approx \frac{mL_A}{2\pi(\alpha-1)}t^{-1} + \mathscr{O}(t^{-3}). && \text{(A.9)} \end{aligned}$$

Similarly, the energy of bosons $\varepsilon_{\mathbf{k}} - \mu$ is always positive and nonzero which implies that the late-time behavior of the energy is $E(x=0,t) = \int dv(\varepsilon(v)-\mu)n(-vt,0) \sim t^{-1}$. Therefore, whenever a system thermalizes with a powerlaw t^{-d}, we will call this ballistic behavior.

To obtain Fig. 1 of the main manuscript, we used a one-dimensional lattice dispersion $\varepsilon_k = -2t\cos k - \mu$. Using this dispersion, in contrast to a free particle dispersion, the $\mathscr{O}(t^{-3})$ term is actually positive which causes the 'bump' in the particle density around $t = 2/J$, see on the right of Fig. 1.

Finally, in the main manuscript we show that the correct quantum description of a hot bosonic system A in a cold bosonic bath displays *diffusive* rather than ballistic behavior. In Fig. A.1 we compare the results of this quantum thermalization to the classical ballistic picture following Eqn. (A.6) at $\beta_B = 5$. The classical picture incorrectly yields a ballistic spread, while the exact quantum computation displays diffusive behavior. The diffusive behavior for cold bosonic baths is therefore a true quantum effect.

Entropy

Entropy plays an important role in quantum many-body physics. Fortunately, the total entropy of the system, which is of course time-independent, is relatively easy to compute using the modular Hamiltonian,

$$S = -\mathrm{Tr}\,\rho(t)\log\rho(t) = \mathrm{Tr}\,\mathscr{M}(t)\rho(t). \tag{A.10}$$

For a free system, this implies that the entropy can be expressed in term of the eigenvalues of the Greens function g_α as[1–3]

$$S = \sum_\alpha \left[-g_\alpha \log g_\alpha + \eta(1+\eta g_\alpha)\log(1+\eta g_\alpha) \right], \tag{A.11}$$

where $\eta = \pm 1$ is the sign for bosons/fermions.

To obtain the entanglement entropy of the subsystem A, we need the reduced density matrix $\rho_A(t) = \mathrm{Tr}_B \rho(t)$. However, for free systems the entanglement entropy can be computed simply by using Eqn. (A.11) where g_α are now the eigenvalues of the Greens function restricted to subsystem A.

Thermalization of relativistic fermions

There is an extensive literature on thermal quenches and thermalization of relativistic particles.[4–7] In one dimension, the Hamiltonian for relativistic fermions is

$$\mathscr{H} = \int dx \left(\psi_R^\dagger(x) i v \partial_x \psi_R(x) - \psi_L^\dagger(x) i v \partial_x \psi_L(x) \right) \tag{A.12}$$

where $\psi_{L,R}(x)$ is the field for left- and right-moving particles, respectively, and $v > 0$ is the Fermi velocity. The energy of the right-movers is $\varepsilon_k^R = vk$ and of left-movers is $\varepsilon_k^L = -vk$. The right-moving nature of the ψ_R field becomes obvious when one expresses the time evolution of the operator,

$$\psi_R^\dagger(x,t) = \int \frac{dk}{2\pi} \psi_{R,k}^\dagger e^{ik(x-vt)}. \tag{A.13}$$

The initial modular Hamiltonian for our hot cloud in A immersed in a bath reads

$$\mathscr{M}_0 = \beta_B \mathscr{H} + (\beta_A - \beta_B) \int_0^{L_A} dx\, h(x) \tag{A.14}$$

Consider only the right-moving particles $h_R(x,t) = \psi_R^\dagger(x,t) i v \partial_x \psi_R(x,t)$ in subsystem A. Under unitary time evolution this segment shifts in its enterity to the right,

$$\int_0^{L_A} dx\, h_R(x,t) = \int_{vt}^{L_A+vt} dx\, \psi_R^\dagger(x) i v \partial_x \psi_R(x) \tag{A.15}$$

Similarly, the left-movers move to the left under time evolution. This means that after a time $t = L_A/v$ there are no remnants of the hot cloud left in the subsystem A. The moment the system A has reached a full causal contact with the bath, it is *instantaneously thermalized*. In Fig. 1 of the main manuscript, middle left, we display the heat profile of such a relativistic system. Notice also that this procedure correctly reproduces the non-equilibrium steady state (NESS) $\mathscr{M} = \beta_+ H + \beta_- P$ as described in Ref.[6].

Decay of modular matrix for nonrelativistic particles

In the main manuscript we showed using the continuum approximation $\varepsilon_\mathbf{k} \approx Jk^2 - \mu$ that the matrix elements of the modular matrix decay ballistically, $\Delta m \sim t^{-d}$. In Fig. A.2 we indeed show that this is the case when computed numerically on large finite size systems. The solid lines are the exact results, and the dashed line is the continuum limit. Note that there are oscillations visible with angular frequency $4J$, which is the result of corrections due to the lattice dispersion $\varepsilon_\mathbf{k} = -2J\sum_{i=1}^d \cos k_i$. The precise shape and amplitude of these oscillations depend on the precise form of the dispersion.

References

1. Vidal, P., Latorre, J.I., Rico, E. & Kitaev, A. Entanglement in Quantum Critical Phenomena. *Phys. Rev. Lett.* **90**, 227902 (2003).
2. Peschel, I. Calculation of reduced density matrices from correlation functions. *J. Phys. A: Math. Gen.* **36**, L205 (2003).
3. Peschel, I. & Eisler, V. Reduced density matrices and entanglement entropy in free lattice models. *J. Phys. A: Math. Theor.* **42**, 504003 (2009).
4. Calabrese, P. & Cardy, J. Time Dependence of Correlation Functions Following a Quantum Quench. *Phys. Rev. Lett.* **96**, 136801 (2006).

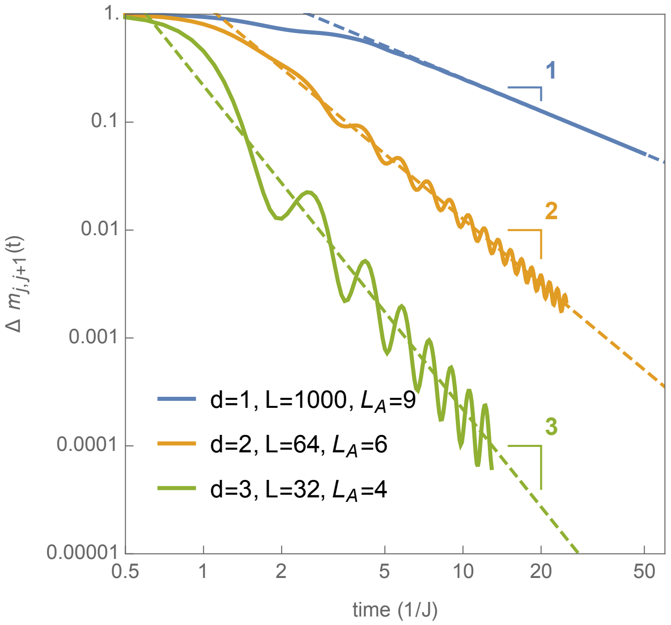

Figure A.2. The decay of the nearest-neighbor modular matrix element $\Delta m_{j,j+1}(t)$ for $j \in A$, for $d = 1, 2$ and 3. The thick solid lines indicate the numerical results, solving Eqn. (11) of the main manuscript exactly on finite size systems. All approach the general formula describing ballistic decay Eqn. (12), here shown as dashed lines.

5. Calabrese, P. & Cardy, J. Quantum quenches in 1+1 dimensional conformal field theories. *J. Stat. Mech.* **06**, 064003 (2016).

6. Bhaseen, M. J., Doyon, B., Lucas, A. & Schalm, K. Energy flow in quantum critical systems far from equilibrium. *Nat. Phys.* **11**, 509 (2015).

7. Lucas, A., Schalm, K., Doyon, B. & Bhaseen, M. J. Shock waves, rarefaction waves, and nonequilibrium steady states in quantum critical systems. *Phys. Rev. D* **94**, 025004 (2016).
